# Inflammatory Caspase Activity Mediates HMGB1 Release and Differentiation in Myoblasts Affected by Peripheral Arterial Disease

**DOI:** 10.3390/cells11071163

**Published:** 2022-03-30

**Authors:** Ricardo Ferrari, Bowen Xie, Edwyn Assaf, Kristin Morder, Melanie Scott, Hong Liao, Michael J. Calderon, Mark Ross, Patricia Loughran, Simon C. Watkins, Iraklis Pipinos, George Casale, Edith Tzeng, Ryan McEnaney, Ulka Sachdev

**Affiliations:** 1 Department of Surgery, University of Pittsburgh Medical Center, Pittsburgh, PA 15213, USA; ferrarirj2@upmc.edu (R.F.); xieb@upmc.edu (B.X.); assafej@upmc.edu (E.A.); morderkt@upmc.edu (K.M.); scotmx@upmc.edu (M.S.); s_unit66@yahoo.com (H.L.); tzenge@upmc.edu (E.T.); mcenaneyrm@upmc.edu (R.M.); 2 University of Pittsburgh Center for Biologic Imaging, Pittsburgh, PA 15213, USA; mjc136@pitt.edu (M.J.C.); mross@pitt.edu (M.R.); loughranp@upmc.edu (P.L.); simon.watkins@pitt.edu (S.C.W.); 3 Department of Surgery, University of Nebraska, Omaha, NE 68198, USA; ipipinos@unmc.edu (I.P.); gpcasale@unmc.edu (G.C.); 4 Department of Surgery, Veterans Affairs Hospital, Pittsburgh, PA 15240, USA

**Keywords:** inflammasomes, caspase-5, myoblasts, peripheral arterial disease

## Abstract

Introduction: We previously showed that caspase-1 and -11, which are activated by inflammasomes, mediate recovery from muscle ischemia in mice. We hypothesized that similar to murine models, inflammatory caspases modulate myogenicity and inflammation in ischemic muscle disease. Methods: Caspase activity was measured in ischemic and perfused human myoblasts in response to the NLRP3 and AIM2 inflammasome agonists (nigericin and poly(dA:dT), respectively) with and without specific caspase-1 or pan-caspase inhibition. mRNA levels of myogenic markers and caspase-1 were assessed, and protein levels of caspases-1, -4, -5, and -3 were measured by Western blot. Results: When compared to perfused cells, ischemic myoblasts demonstrated attenuated MyoD and myogenin and elevated caspase-1 mRNA. Ischemic myoblasts also had significantly higher enzymatic caspase activity with poly(dA:dT) (*p* < 0.001), but not nigericin stimulation. Inhibition of caspase activity including caspase-4/-5, but not caspase-1, blocked activation effects of poly(dA:dT). Ischemic myoblasts had elevated cleaved caspase-5. Inhibition of caspase activity deterred differentiation in ischemic but not perfused myoblasts and reduced the release of HMGB1 from both groups. Conclusion: Inflammatory caspases can be activated in ischemic myoblasts by AIM2 and influence ischemic myoblast differentiation and release of pro-angiogenic HMGB1. AIM2 inflammasome involvement suggests a role as a DNA damage sensor, and our data suggest that caspase-5 rather than caspase-1 may mediate the downstream mediator of this pathway.

## 1. Introduction

Peripheral arterial disease (PAD), which can cause a clinical spectrum of diseases ranging from relatively benign intermittent claudication to limb-threatening ischemia is prevalent in >20% of patients over the age of 70 [1]. Treatments include medical optimization, endovascular interventions, and open surgical approaches to revascularization. Amputation can be a significant risk if revascularization is not possible [1]. In addition to suffering with nonhealing wounds, rest pain, or pain from exercise-induced ischemia, people with PAD also have muscle dysfunction [2,3,4]. Research suggests that sarcopenia is a consequence of PAD, reflective of chronic ischemia-induced damage [5]. Myopathy with concomitant biochemical changes has also been associated with PAD, resulting in weakness [2,3,4,6]. Despite this information, research into muscle satellite cell (MuSC)-derived myoblast function in ischemic skeletal muscle tissue is limited. Myoblast functionality is required for regenerative capacity, which makes it an important area for PAD-related research. MuSC are located in specialized compartments between the sarcolemma and basal lamina, and respond to injury by differentiating into myoblasts, proliferating and fusing to each other and to injured myofibers [7]. We have shown that inflammatory caspases-1 and -11, which are cleaved and thus activated by inflammasome oligomerization, play a role in muscle recovery following ischemic injury in mice. However, the role of inflammatory caspases in mediating injury and repair in human muscle is less well described. Inflammatory caspases in humans include caspase-1, the canonical mediator of nucleotide-binding oligomerization domain, leucine-rich repeat, and pyrin domain-containing (NLRP3) and are absent in melanoma (AIM2) inflammasomes [8]. Humans also have caspases-4 and -5, which are homologs of murine caspase-11 [9] and mediate noncanonical inflammasome activation. Caspase-5 has been less studied than caspase-4 but has been shown to promote inflammatory cytokine release from monocytes without needing a priming step typically required for inflammasome activation [10]. We have previously shown that the nuclear protein and damage-associated molecular pattern (DAMP) high-mobility group box 1 (HMGB1) is released from myoblasts in an inflammatory caspase-1-dependent [11]. We have also shown that HMGB1 promotes regeneration and angiogenesis in a murine model of hindlimb ischemia and that lack of both HMGB1 as well as inflammatory caspases worsens recovery from ischemic muscle injury [12]. Therefore, we sought to determine the role of inflammatory caspases in ischemic muscle from PAD patients. We hypothesized that in PAD, inflammatory caspase activity mediates HMGB1 release and myocyte fusion, which is critical for muscle regeneration following injury. 

## 2. Materials and Methods

### 2.1. Human Subjects for Myoblast Isolation

All protocols involving human tissue were reviewed by the Institutional Review Boards (IRB) of participating institutions. Human subject research was performed in accordance with the ethical guidelines put forth by the Declaration of Helsinki and approved by the IRB of the University of Pittsburgh (Study19050272) and Omaha VA (#00086). Ischemic myoblasts were obtained from proximal and distal tibialis anterior (TA) muscle harvested at the time of amputation for severe non-reconstructable PAD. Perfused myoblasts were obtained from non-ischemic segments of *vastus medialis*/*lateralis* and from commercial sources (Cook MyoSite Company, Pittsburgh, PA, USA). Blood pressure indices to the brachial artery (ABI), as well as pulse volume recordings (PVR), were used to assess perfusion to a particular region. ABI < 0.9 connoted PAD, and ABI < 0.7 was consistent with moderate to severe PAD. PVR with slow rise time, flat or rounded peak, and absent dicrotic notch suggested stenosis of the artery just above the measurement, resulting in muscle ischemia to the segment below it [13]. Subjects with end stage renal disease on dialysis were not included.

### 2.2. Human Subjects for Core Needle Biopsies

Patients undergoing surgery for PAD-associated intermittent claudication (IC) and critical limb ischemia (CLI), as well as those undergoing surgery for non-PAD-related vascular diagnoses (varicose veins and abdominal aortic aneurysm), were recruited as described previously [2]. The diagnosis of PAD was established with a combination of the following factors (1) the presence of characteristic symptoms and signs of IC, rest pain, ischemic ulceration, or gangrene in the affected limb; (2) absence or diminution of pedal pulses, dependent rubor, trophic skin changes, ischemic ulcers, and gangrene; (3) diminished ABI and/or great toe pressure in the symptomatic limb or limbs as determined by segmental pressures measurement and ABI < 0.9, and angiography (if available) demonstrating arterial occlusive disease depending on the indication for the primary operation. To be eligible to serve as a control, the patient had to have no history of PAD with ABI > 0.9 in the limb sampled. 

### 2.3. Myoblast Harvest

Approximately 2 g of muscle was cleaned of connective tissue and fat inside a sterile culture hood, transferred to a 10 cm dish containing pH-balanced buffer, and minced using sterile scissors. The slurry was washed and transferred to 50 mL tubes and centrifuged for 5 min. The resulting pellet was subsequently homogenized using dispase II (1.2 U/mL; Roche Diagnostics, Basel, Switzerland, #11088882001) and collagenase D (5 mg/mL; Sigma, St. Louis, MO, USA # D4693-1G). Tubes were placed at 37 °C and 5% CO_2_ incubator to initiate isolation of the slowest-adhering fraction. Supernatants containing the MuSC were sequentially transferred to new plates and allowed to incubate for 7 days, allowing most cells to attach and achieve approximate confluence for subsequent cell sorting. Cells were maintained in a low-glucose medium containing 16% fetal bovine serum (FBS; Gibco, Waltham, MA, USA, #16140071), fetuin, 7.5% bovine serum albumin (BSA; Sigma, #A8412), dexamethasone (Sigma, #D8893), gentamicin (Gibco, #15750-060), human epidermal growth factor (hEGF; Gibco, #PHG0311), and fungizone (Gibco, #15290-018), which was replaced every 48 h to minimize cell disruption. The slowest adhering fractions were then removed and subjected to magnetic assisted cell sorting (MACS; Miltenyi Biotech, zBergish Gladbach, Germany, #130-090-312; MS Columns, #130-042-201) with CD56-conjugated magnetic beads (Miltenyi Biotech, #130-050-401) [14]. An outline of tissue processing is demonstrated in Figure S1.

Myoblast isolation was confirmed using immunofluorescence, which previously demonstrated this population to be negative for the stem cell marker Pax7. Staining was performed by seeding 2 × 10^5^ myoblasts on chamber slides (MatTek, Ashland, MA, USA, #P35GCol-1.5-10-C) in media supplemented with 16% FBS. The cells were washed, fixed, permeabilized, and incubated with primary antibodies to MyoD1 (Abcam, Waltham, MA, USA, #ab64159; 1:250) in 1% bovine serum albumin solution (BSA) as well as TE-7 (Milipore, Burlington, MA, USA, #3112589; 1:100). To assess cell viability, a total 1 × 10^4^ cells were plated in 96-well plates. After 24 h, CellTiter-Glo Luminescence cell viability assay reagent (Promega, Madison, WI, USA, # G7570) was added to each well, and the wells were incubated at RT for 10 min. Relative luminescence was measured using a Biotek microplate reader (BioTek; Winooski, VT, USA, Synergy LX Multi-Mode Microplate Reader). Measurements were made in triplicate. 

### 2.4. Core-Needle Biopsies

Patients undergoing core-needle biopsy were prospectively recruited, and biopsies were performed under anesthesia. In brief, the area on the skin over the muscle group to be sampled was injected with local anesthetic. The biopsies were performed using a 6 mm Bergstrom needle. Muscle samples were fixed in ice-cold methacarn, embedded with paraffin, and serially sectioned for histologic analysis [2].

### 2.5. Myogenicity Studies

Myoblast differentiation—Twenty-thousand cells were seeded in chamber slides and allowed to differentiate over 5 days in culture media supplemented with 2% FBS, which promotes myoblast differentiation [15]. After washing, cells were again fixed, permeabilized, and incubated with antibodies to MF20 (10 μg/mL; R&D systems, Minneapolis, MN, USA, #MAB4470) to identify myosin-heavy chain 2 (MCH2) as well as DAPI 1:1000 to identify nuclei. Cells were imaged using an Olympus Fluoview 1000 confocal microscope at 20× magnification. Myocyte fusion index (MFI) was calculated by dividing the total number of nuclei in the image by the total number of nuclei within multinucleated myocytes (>2 nuclei). Four or five images were taken per treatment. 

Incucyte^®^ live-cell imaging—Cell proliferation, differentiation, and apoptosis was assessed over time using Incucyte^®^ Live Cell Imaging (IncuCyte^®^ Live-Cell Analysis System SX5). Myoblasts were plated in a pre-coated 96-well clear bottom plate (10,000 cells per well; Corning, NY, USA, #3610) overnight and supplemented with 16% fetal bovine serum. For proliferation, Incucyte NucLight Rapid Red Reagent (Sartorius, Gottingham, Germany, #4717) diluted in complete medium (5 μM final concentration) was added to each well. To assess apoptosis, caspase-3/7 green reagent (Sartorius, #4440) diluted 1:1000 in complete medium (5 μM final concentration) was added in each well. Measurements were performed in duplicate.

The plates were placed into the IncuCyte^®^ Live-Cell Analysis System SX5 and warmed to 37 °C for 30 min prior to scanning. Cells were imaged in phase contrast and red or green fluorescence with a 20× objective every 2 h for a total of 120 h (proliferation and differentiation) and 72 h (apoptosis). The software Incucyte^®^ Controller Version 2020A was used to quantify cell confluence and total amount of caspase 3/7 per image. For tube formation, network length, tube number, and tube width were calculated using the Incucyte^®^ Angiogenesis Analysis Software Module (Cat. No. 9600-0011), which was chosen due to its ability to quantify fusion and tube forming behavior. Measurements were performed in duplicate.

### 2.6. Real-Time Polymerase Chain Reaction (RT-PCR)

MuSC were plated on 12-well plates and allowed to proliferate for 24 h or differentiate for a total 5 and 10 days, as described above. Total RNA was extracted using an RNeasy Mini kit (Qiagen, Germantown, MD, USA, #74134) following the manufacturer’s protocol. The reverse polymerase transcription was performed using reverse transcription reagents (Applied Biosystems, Bedford, MA, USA, #4374966) according to the manufacturer’s instructions. TaqMan probes included MyoD1 (Hs00159528_m1), myosin heavy chain 2 (Myh2; Hs00430042_m1), insulin growth factor-1 (IGF1; Hs01547656_m1), and caspase-1 (Casp1; Hs00354832_m1; Applied Biosystems). MyoD1, MYH2, IGF1, and Casp1 expression levels were determined by quantitative Real Time-qPCR on an Applied Biosystem 7900 HT Fast Real-Time PCR System using a commercially available TaqMan PCR master mix. The 2^−ΔΔCT^ method was used to quantify the relative expression levels of the genes by ExpressionSuite Software Version 1.0.3 (Life Technologies, Waltham, MA, USA). Relative gene expression was calculated first by correcting each gene cycle threshold (CT) by the average CT value for housekeeping genes that were unchanged across groups (GAPDH and CSNK2A2). Subsequently, the log 2-fold change (FC) was calculated relative to the number of myotubes differentiated at 3 time points (1, 5, and 10 days) from each experimental group. mRNA expression levels for all samples were normalized to the housekeeping genes. 

### 2.7. Immunohistochemistry and Cytochemistry

Sections obtained from core needle biopsies were deparaffinized and incubated with primary antibodies to total caspase-1 (Invitrogen, Waltham, MA, USA, #MA5-32909), dystrophin (Abcam, #ab15277), and Cy3-conjugated antibody to smooth muscle actin (Sigma, C6198). DAPI was used to identify nuclei. The fluorophore-conjugated secondary antibodies were donkey anti-rat against caspase-1 (Life Technologies #, A21208) and donkey anti-rabbit against dystrophin (Jackson Immunoresearch, Westgrove, PA, USA, # 711-176-152). Images were taken using a Nikon A1 confocal microscope at a magnification of 20 × 2 z. Using the ImageJ analysis program, images were color-separated, and a threshold was applied to calculate the integrated density of caspase-1 expression for each 20× section. Four or five images were taken per section. MuSC were also subjected to immunocytochemistry for detection for AIM2. A total of 2 × 10^5^ MuSC were plated on MatTek dishes, washed, fixed, permeabilized, and blocked before being incubated with primary antibodies to AIM2 (Abcam, # ab204955, 1:200 in 1% BSA) overnight at 4 C. Cells were washed and incubated with goat anti-mouse IgG (Alexa Fluor^®^ 488, Abcam, #ab150117) and counterstained with DAPI and phalloidin (1:1000 in PBS, Abcam, #176759) for 30 min each. Cells were imaged using the OlympusIX81 inverted microscope at 20× magnification. Four to five images were taken per treatment. 

### 2.8. Caspase-1 Activity Fluorometric Assay

MuSC harvested from patients as well as commercial myoblasts were cultured for 24 h or differentiated for five days and lysed for evaluation of caspase-1, -4, and-5 activity (Z-WEHD-aminoluciferin, Promega, #G9951) in the presence and absence of specific inhibitors to caspase-1 (Ac-YVAD-cho) or to broader caspase activity (Z-VAD-FMK). The caspase-1 inhibitor has previously been showed not to affect caspase-4 and -5 (PMID 16465268). Cells were additionally treated with poly(dA:dT)/LyoVec (Invivogen, #TLRLPATC) and nigericin (Sigma, #SML1779) to assess activation of AIM2 and NLRP3 inflammasomes, respectively [8,16]. Measurements were performed in triplicate.

### 2.9. Western Blot

MuSC were lysed in phenylmethanesulfonyl fluoride (PMSF), and protein content was quantified with bicinchoninic acid, loaded onto SDS-PAGE gels, and transferred onto nitrocellulose membranes as described previously [12]. Immunoblots were probed with antibodies to caspase-1 (Cell Signaling, Danvers, MA, USA, #2225S), caspase-5 (Cell Signaling, #46680S), caspase-3, gasdermin D (Abcam, #210070), and actin (Cell Signaling, #3700T). Chemiluminescence was used to detect protein expression. ImageJ analysis program [17] was for semi-quantitative analysis of each protein normalized to actin. 

### 2.10. Statistical Analysis

Data were assessed for normality and equal variance. Comparisons between 3 or more groups were performed by one-way ANOVA, with Tukey’s *t*-test for multiple comparisons. The cutoff for statistical significance was set at *p* < 0.05. All statistical analyses were performed on Graph Pad Prism version 8 (Graph Pad Software, Inc San Diego, CA, USA).

## 3. Results

### 3.1. Myoblasts in PAD Were Viable but Their Differentiation Capacity Was Reduced

#### 3.1.1. Myoblasts Can Be Harvested from Ischemic Muscle Tissue 

**Figure 1:**
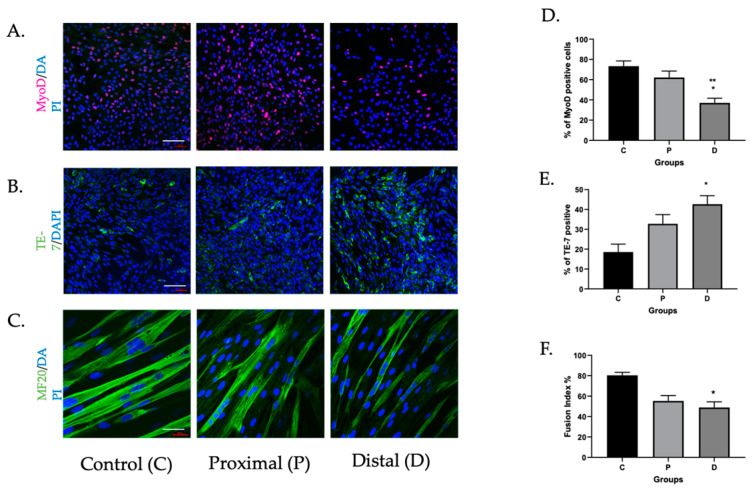
Myoblasts harvested from ischemic muscle had attenuated differentiation compared to those from perfused segments. Myoblasts harvested from proximal (P) and distal (D) segments of muscle affected by PAD (N = 4) were compared to those from perfused controls (C) (N = 2) for markers of muscle or fibroblast origin, and differentiated over five days. (**A**,**D**) MyoD (* *p* < 0.03, ** *p* < 0.002, distal vs. proximal and control, respectively); (**B**,**E**) TE-7 (* *p* < 0.03, distal vs. control). Myocyte fusion index (MFI); (**C**,**F**) calculated by dividing the number of nuclei within multinucleated MF20-positive cells (>2) by the total number of nuclei (* *p* = 0.03, ischemic vs. control). Images are representative. Scale bar = 10 μM.

The mean ABI in patients from whom myoblasts were harvested was 0.52 ± 0.19, confirming significant arterial insufficiency in the population. Both ischemic and perfused myoblasts had similar viability after 24 h (*p* = ns). Myoblasts harvested from proximal tibialis anterior (TA) segments demonstrated comparable levels of MyoD positivity to those obtained from perfused controls, confirming myogenic origin (Figure 1A,D). Distal TA samples demonstrated a higher percentage of fibroblasts (TE-7+) than in proximal or control samples (Figure 1B,E), although MyoD-positive cells predominated. To evaluate the impact of PAD on myoblast differentiation, we measured myoblast fusion index (MFI) for each group of cells, allowing cells to differentiate over five days in optimized culture. Proximal and distal TA MuSC had significantly lower MFI than non-PAD control cells (Figure 1C,F). These data suggest attenuation of the critical role of myoblasts to fuse together to restore muscle mass in ischemic myoblasts. 

**Figure 2:**
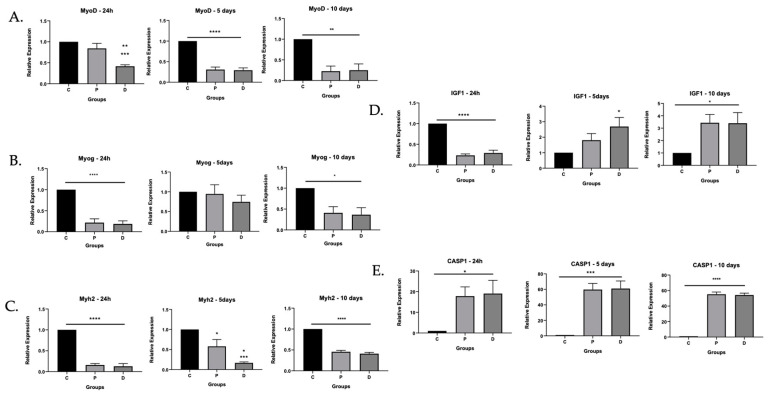
Differential mRNA expression suggested decreased myogenic differentiation and increased inflammatory caspase in ischemic myoblasts. Perfused (N = 2) and ischemic myoblasts (N = 3) were allowed to proliferate for 24 h and to differentiate for 5 or 10 days. RNA was extracted and probed for markers associated with muscle differentiation as well as inflammatory caspase-1. (**A**) MyoD; (**B**) myogenin; (**C**) Myh2; (**D**) IGF1; (**E**) caspase-1; * *p* < 0.05, ** *p* < 0.01, *** *p* < 0.001, **** *p* < 0.0001.

Given the diminished ability for ischemic cells to differentiate, we evaluated gene expression for markers associated with muscle identity, differentiation, and growth. We performed qRT-PCR and evaluated mRNA expression at 24 h and at 5 and 10 days of differentiation of MyoD, the aforementioned marker of muscle satellite cell origin; myogenin, a transcription factor that modulates terminal differentiation and exits from the cell cycle [18]; Myh2 [19], which increases with differentiation; insulin growth factor (IGF), which is an important modulator of regeneration of skeletal muscle [20]; and caspase-1, which can mediate inflammatory and noninflammatory forms of cell death [9]. The data confirmed patterns of expression consistent with deterred differentiation in ischemic cells (Figure 2). MyoD mRNA was diminished in ischemic cells taken from distal TA. Following differentiation, proximal and distal TA myoblasts had attenuated MyoD and myogenin in contrast to healthy donor cells (Figure 2A,B). Similarly, Myh2, which indicates terminal differentiation, was attenuated in proximal and distal TA after 5 and 10 days of differentiation but was maintained in healthy donor control cells (Figure 2C). As a key driver of differentiation, IGF-1 mRNA would be expected to be high before differentiation and low after differentiation. As expected, IGF-1mRNA was high in healthy donor control cells after 24 h and diminished after 5 and 10 days of differentiation. However, in proximal and distal TA, IGF mRNA remained elevated after 5 and 10 days of differentiation (Figure 2D), suggesting deterred differentiation. Caspase-1 mRNA was also significantly higher in proximal and distal TA myoblasts after 5 and 10 days of differentiation but was nearly undetectable in control cells (Figure 2E), suggesting activation of inflammatory pathways in ischemic conditions. 

#### 3.1.2. Inflammatory Caspase Activity in Ischemic Myoblasts Responded to AIM2 Agonists

Our results demonstrated elevated caspase-1 mRNA in ischemic proximal and distal TA myoblasts during differentiation. To confirm whether this might have clinical correlation in patients with PAD, we performed immunohistochemical staining on sections obtained from gastrocnemius muscle during operations for non-PAD related illness (control), as well as for a PAD diagnosis (IC or CLI). Results are shown in Figure S2. Caspase-1 protein expression was significantly higher in PAD muscle segments harvested from patients with IC, confirming that caspase-1 expression may be relevant in clinical disease. 

**Figure 3:**
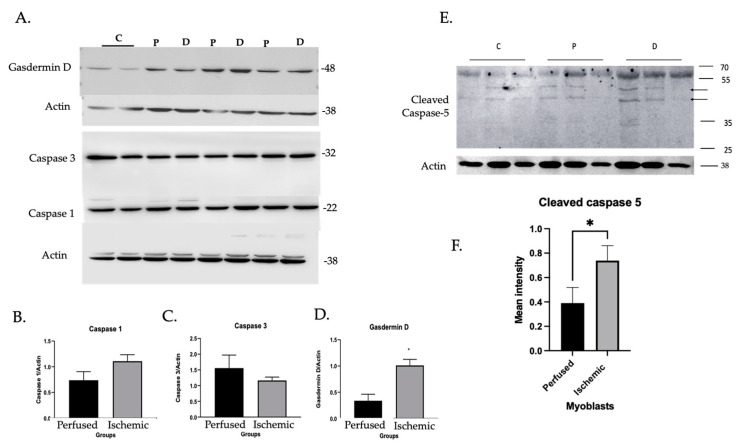
Cleaved caspase-5 and gasdermin D were elevated in ischemic myoblasts. Western blot was performed in perfused (N = 2) and ischemic myoblasts (N = 6) to determine the expression of caspase-1, caspase-3, gasdermin D, and cleaved caspase-5. Actin was used as a loading control. Normalization. kDa are shown. Relative density of each band compared to that of actin and calculated using ImageJ. (**A**) Western blot image of caspase-1, caspase-3, and gasdermin D. (**B**) Quantification of caspase-1 (NS). (**C**) Quantification of caspase-3 (NS). (**D**) Quantification of gasdermin D (* *p* < 0.03). (**E**) Cleaved caspase-5 Western blot with arrows denoting cleaved products. (**F**) Quantification of cleaved caspase-5 (*p* < 0.03, N = 3 perfused, 6 ischemic).

Given this information, we further evaluated expression and activity of inflammatory caspases in myoblasts including caspases-1, -4, and -5. Pyroptotic roles of inflammatory caspases depend on gasdermin D (GSDMD), a pore-forming protein that mediates cell lysis and release of proteins such as HMGB1 [21]. However, caspase-1 can also promote apoptosis through caspase-3 effector activity [22]. Thus, we also probed for caspase-3 and GSDMD. Caspases-1 and -3 were expressed in both ischemic and perfused cells. GSDMD was elevated in ischemic cells (Figure 3A–D). Importantly, caspase-5 and cleaved forms suggestive of activation were expressed at higher levels in ischemic compared with perfused cells [23]. No signal was detected for caspase-4 (not shown). 

**Figure 4:**
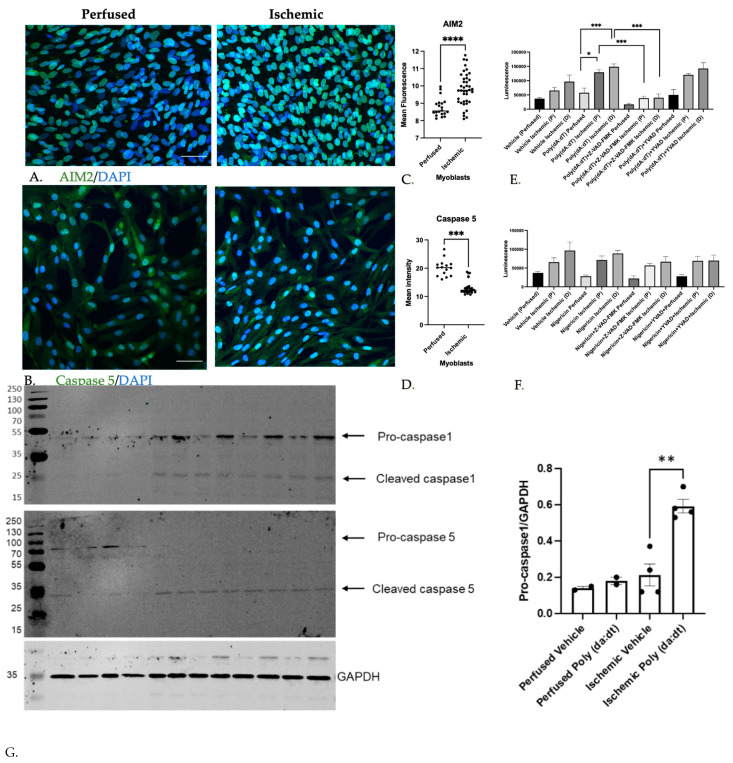
AIM2 expression was elevated and promoted inflammatory caspase activity in ischemic myoblasts. Expression of AIM2 and caspase-5 (total) were calculated in perfused and ischemic myoblasts using immunofluorescence. DAPI was used to detect nuclei, and mean intensity per cell was calculated using ImageJ for a total of 5 images per cell group (N = 2 control, N = 3–4 ischemic). (**A**,**C**) AIM2 immunofluorescence and quantification (**** *p* < 0.0001); (**B**,**D**) caspase 5 (*** *p* < 0.001). Luminescence assays were used to detect inflammatory caspase activity induced by caspase-1, -4, and -5 in perfused and ischemic cells with and without either caspase-1-specific or additional pan-caspase inhibition. Cells were additionally exposed to (**E**) poly(dA:dT), an AIM2 agonist (* *p* < 0.05, *** *p* < 0.001) and (**F**) nigericin, an NLRP3 agonist. Scale bar = 10 μM. (**G**) Detection of pro-and cleaved caspase 1 and caspase 5 by Western blots performed with perfused and ischemic myoblast with and without poly(dA:dT). ** *p* < 0.002 (N = 2 perfused, 4 ischemic).

We also confirmed caspase-5 expression as well as AIM2 expression using immunofluorescence. Isolated myoblasts from perfused and ischemic segments demonstrated higher levels of AIM2 (Figure 4A,C). Full length caspase-5 level was decreased (Figure 4B,D), corresponding with the higher cleaved forms demonstrated on Western blot. We then sought to determine whether caspase activity was also elevated. We performed a fluorometric inflammatory caspase activity assay on whole-cell lysates after 5 days of differentiation that utilized a substrate cleaved by both caspase-1 and caspase-5 (Z-WEHD-aminoluciferin; Promega) [23]. Cells were additionally exposed to specific inhibitors of caspase-1 (YVAD) as well as an inhibitor that additionally blocked caspases-3 through -10 (Z-VAD-FMK). Cells were activated with poly(dA:dT) and nigericin, activating AIM2 and NLRP3 inflammasomes, respectively [24,25]. Results are shown in Figure 4E,F. Only ischemic myoblasts responded to poly(dA:dT) with a significant increase in inflammatory caspase activity (Figure 4E). In contrast, inflammatory caspase activity did not increase with exposure to the NLRP3 agonist nigericin in either perfused or ischemic myoblasts (Figure 4F). Inflammatory caspase activity was attenuated only with the pan-caspase inhibitor Z-VAD-FMK but not with the specific caspase-1 inhibitor YVAD. Taken together, the fluorometric assay, immunoblot, and immunofluorescence reflected increased caspase-5 activity in ischemic myoblasts. To further confirm an increase in caspase expression and cleavage of caspases-1 and -5 in ischemic cells with and without AIM2 activation, Western blot was also performed. Effects of AIM2 activation with poly(dA:dT) were only seen in ischemic cells (Figure 4G). 

#### 3.1.3. Caspase Inhibition Reduced Myocyte Differentiation in Ischemic Myoblasts 

**Figure 5:**
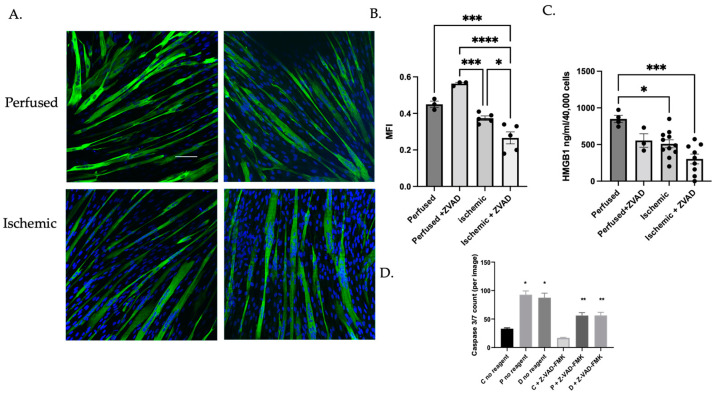
Pan-caspase inhibition attenuated myocyte differentiation in ischemic myoblasts and reduced HMGB1 release during myocyte fusion. Perfused and ischemic (proximal and distal) myoblasts were induced to differentiate in the presence or absence of Z-VAD-FMK. (**A**) Cells were immunostained with antibodies against MF20, which detects myosin heavy chain 2, a marker of differentiation. Nuclei were detected using DAPI, and the myocyte fusion index (MFI) was calculated. (**B**) MFI in perfused and ischemic cells with and without Z-VAD-FMK (* *p* < 0.05, *** *p* < 0.001, **** *p* < 0.001). (**C**) Supernatants were collected and subjected to ELISA to determine HMGB1 concentration (* *p* < 0.03, ** *p* < 0.001). (**D**) Incucyte live cell imaging was performed to assess expression of apoptotic markers over time, with and without Z-VAD-FMK, and final count was assessed after five days (* *p* < 0.03, ** *p* < 0.01). Scale bar = 10 μM.

We showed that myoblasts from ischemic regions of skeletal muscle demonstrated attenuated differentiation and increased caspase activity induced by AIM2 inflammasome activation. Caspase activity is also important for release of HMGB1 from differentiating myoblasts. While HMGB1 is a pro-inflammatory signal, it also promotes muscle regeneration [26]. We therefore asked whether blocking caspase activity would influence capacity of myoblasts to differentiate, potentially as a function of reduced HMGB1 release. To answer this question, we treated myoblasts isolated from perfused or ischemic regions with Z-VAD-FMK and assessed myocyte fusion. Exposure to Z-VAD-FMK reduced myocyte fusion index in ischemic but not in perfused cells (Figure 5A,B). 

#### 3.1.4. MuSC Released HMGB1 in a Caspase-Dependent Manner

We then sought to determine whether inflammatory caspase activity in human myoblasts could mediate release of HMGB1 from cells, which we had demonstrated in mouse myoblasts [11,12,26,27]. Since ischemic myoblasts expressed increased levels of pore-forming GSDMD, we hypothesized that HMGB1 release will also be higher from ischemic myoblasts. We measured HMGB1 levels in cell culture supernatants from differentiating myoblasts by ELISA. Strikingly, myoblasts from ischemic muscle released less HMGB1 during differentiation than those from perfused segments (Figure 5C). Pan-caspase inhibition with Z-VAD-FMK significantly attenuated HMGB1 release across all groups. To assess the ability of the pan-caspase inhibitor to block caspase activity, we measured caspase-3/7 expression using live cell imaging. Apoptosis was attenuated by Z-VAD-FMK (Figure 5D). Taken together, these data suggest that in both ischemic and nonischemic myoblasts that were stimulated to differentiate, HMGB1 was released by a caspase-mediated process. 

## 4. Discussion

In this study, we aimed to identify factors within chronically ischemic myoblasts that might provide therapeutic targets to improve their function within the ischemic limb, improving quality of life and preventing limb loss. Our study has some important findings that address this goal. First, myoblasts were successfully harvested from densely ischemic limbs that were subjected to amputation. While those segments exposed to higher degrees of ischemia (i.e., in the distal crural segments) showed some evidence of fibroblast infiltration, myoblasts still predominated in the population. Second, myoblasts that were harvested from ischemic muscle were viable and able to proliferate in culture conditions. However, when compared to cells from healthy donors and from perfused muscle segments, myoblasts harvested from chronically ischemic muscle segments had a diminished ability to differentiate. Differentiation is a key element of muscle regeneration after injury [28]. Thus, the diminished ability of MuSC to differentiate may play a role in weakness in PAD. 

Myoblasts harvested from ischemic muscle segments also demonstrated evidence of caspase-1 upregulation by mRNA assessment and had elevated inflammatory caspase activity particularly to agonists of the AIM2 inflammasome. Myocytes are not inflammatory cells but appear to harness elements of the inflammasome machinery during differentiation. Reports of inflammasome function in muscle has focused on NLRP3 and a potential role in cardiomyocytes rather than skeletal muscle [29,30]. Specifically, the NLRP3 inflammasome has been linked to clinical atrial fibrillation, presumably reflecting an inflammatory state [30]. 

In contrast, the AIM2 inflammasome is a compelling target to study in tissues that regenerate. Recently, AIM2 has been shown to play a protective role in neurodevelopment due to its detection of cytosolic DNA and DNA damage. Specifically, loss of the AIM2 inflammasome resulted in behavioral abnormalities in mice. This phenotype was due to a loss of programmed inflammatory cell death governed by AIM2 in the setting of DNA damage [31]. Thus, AIM2 provided a mechanism for cells with DNA damage to undergo lysis, preventing faulty replication. Furthermore, cytosolic HMGB1 has been shown to interact with AIM2 during redox stress in hepatocytes, leading to potential protective roles [32]. It is not known whether the mechanism of “quality control” or AIM2 association with HMGB1 mobilization is relevant in muscle. 

We showed that inflammatory caspases may support myoblast differentiation in PAD, and our data suggest that inflammasome activation may be important for myocyte fusion during ischemia. Specifically, we showed that MuSC in PAD express caspase-5, a mediator of noncanonical inflammasome activation. Caspases-4 and -5 are analogs of mouse caspase-11 [33]. In our mouse model of femoral artery ligation, loss of both caspases-1 and -11 resulted in poor regeneration in ischemic muscle [11]. Our current results also suggest a protective role for inflammatory caspases in human myoblast differentiation, particularly when experiencing ischemic injury. This may even occur independent of inflammasome activity. For example, caspase-5 mediates activation of human monocytes in the absence of AIM2 or Nod-like receptor engagement [34]. Caspase-5 has also been shown to mediate cytokine release from macrophages without concomitant cell death, unlike activation of caspase-4 in which cell death is prominent [35]. The fact that AIM2 expression was higher in ischemic myoblasts suggests that it may be playing a role in the face of injury, perhaps by responding to DNA damage or cytosolic DNA. Engagement of cells with poly(dA:dT) showed that AIM2 is not just present, but is also able to be activated in ischemic cells. 

Despite an apparent dependence on caspase activity to support HMGB1 release and differentiation, ischemic cells demonstrated decreased HMGB1 release when cells were prompted to fuse. Furthermore, inhibition of caspase activity not only deterred fusion in ischemic cells, but significantly decreased HMGB1 release. One interpretation is that release of HMGB1 is necessary for differentiation by paracrine effects, and its release is mediated by inflammatory caspase activation. Morever, the baseline deficiency of HMGB1 release could be due to an “exhausted” state, where cells have already released HMGB1 through caspase-mediated activity. We showed here that caspase-1 expression was higher in intermittent claudication, but undetectable in densely ischemic tissue affected by critical limb ischemia, which may also reflect similar state of cell exhaustion which is a hallmark of cellular senescence [36]. Another possible explanation for why HMGB1 release was reduced in ischemic cells is that its translocation is limited [37]. We showed very prominent nuclear HMGB1 staining in ischemic muscle affected by CLI, which would support this idea [38]. 

Despite a noticeable deficit in differentiation in ischemic myoblasts, our data also suggested that the drive to differentiate might have been maintained in those same cells. Insulin growth factor, which is upregulated during muscle growth and differentiation, remained elevated in myoblasts from PAD even after five days of culture, while cells from healthy donors downregulated IGF expression over the same period of time (Figure 2). These differences negatively correlated with Myh2, a marker of terminal differentiation that was elevated in healthy donor but not PAD cells. IGF-1 is a key regulator of muscle satellite cell growth and differentiation [20,39]. Its persistence may be due to lack of feedback signals to “turn off” [40].

There are some important limitations to our study. Patient comorbidities were not assessed as potential contributors to muscle dysfunction. Patients with PAD often have concomitant diabetes, hypertension, and chronic kidney disease, which may be confounding. This current study was not powered to detect the influence of those factors but future studies in our laboratory aim to do so. 

In summary, we demonstrate that while myoblasts harvested from PAD patients are viable and able to proliferate, they lack an ability to differentiate to the same degree as non-ischemic cells. This deficiency occurs even after cells are optimized ex vivo with nutrients and appropriate oxygen concentration. These findings correlate with a robust inflammatory caspase activity within the myoblasts themselves which appear to play a role in mediating differentiation in ischemic cells. It is possible that noninflammatory cells co-opt these systems typically seen in inflammatory cells as a mechanism to allow for release of DAMPs that promote regeneration. While our studies showed that HMGB1 release is reduced in PAD, this might be because stores have been exhausted, mobilization is deterred, or both. Additionally, chronic ischemia might lead to irreversible changes in the ability of MuSC to differentiate and might argue for earlier treatment even in presumably more benign presentations such as IC.

## 5. Conclusions

Our data suggest that ischemia from PAD alters the myogenic potential of myoblasts and that inflammasome activation may be a compensatory mechanism for repair. We suspect that inflammasome activation may ultimately be protective in ischemic cells by promoting release of pro-regenerative HMGB1, as well as by serving in cellular quality control.

## Data Availability

Data in this report can be made available upon reasonable written request.

## Supplementary Materials

The following supporting information can be downloaded at https://www.mdpi.com/article/10.3390/cells11071163/s1, Figure S1: Myoblast Harvest—graphic demonstrating source of muscle tissue from human samples and processing techniques to isolate myoblasts. Figure S2: Caspase-1 expression was elevated in patients with intermittent claudication.
