# Supplementary material for: Inflammatory Caspase Activity Mediates HMGB1 Release and Differentiation in Myoblasts Affected by Peripheral Arterial Disease
Source: Cells. 2022 Mar 30;11(7):1163. doi: 10.3390/cells11071163 (PMC8997414; doi:10.3390/cells11071163)
Supplement: Supplementary file 1 [file cells-11-01163-s001.zip › Supplemental Figure S1.pptx]

## Slide 1
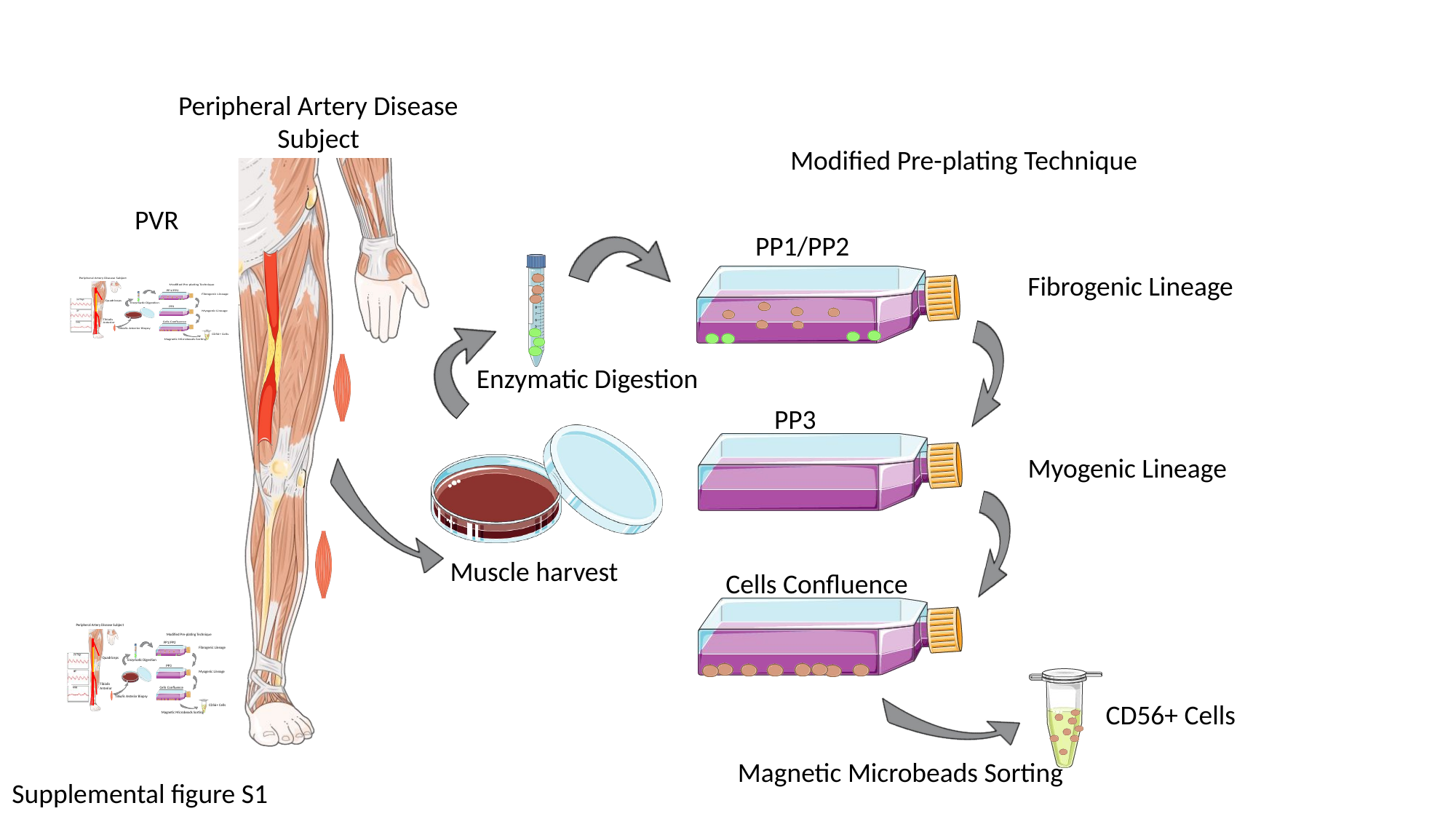

Peripheral Artery Disease
Subject
Modified Pre-plating Technique
PVR
PP1/PP2
Fibrogenic Lineage
Enzymatic Digestion
PP3
Myogenic Lineage
Muscle harvest
Cells Confluence
CD56+ Cells
Magnetic Microbeads Sorting
Supplemental figure S1
