# Supplementary material for: Inflammatory Caspase Activity Mediates HMGB1 Release and Differentiation in Myoblasts Affected by Peripheral Arterial Disease
Source: Cells. 2022 Mar 30;11(7):1163. doi: 10.3390/cells11071163 (PMC8997414; doi:10.3390/cells11071163)
Supplement: Supplementary file 1 [file cells-11-01163-s001.zip › Supplemental Figure S2.pptx]

## Slide 1
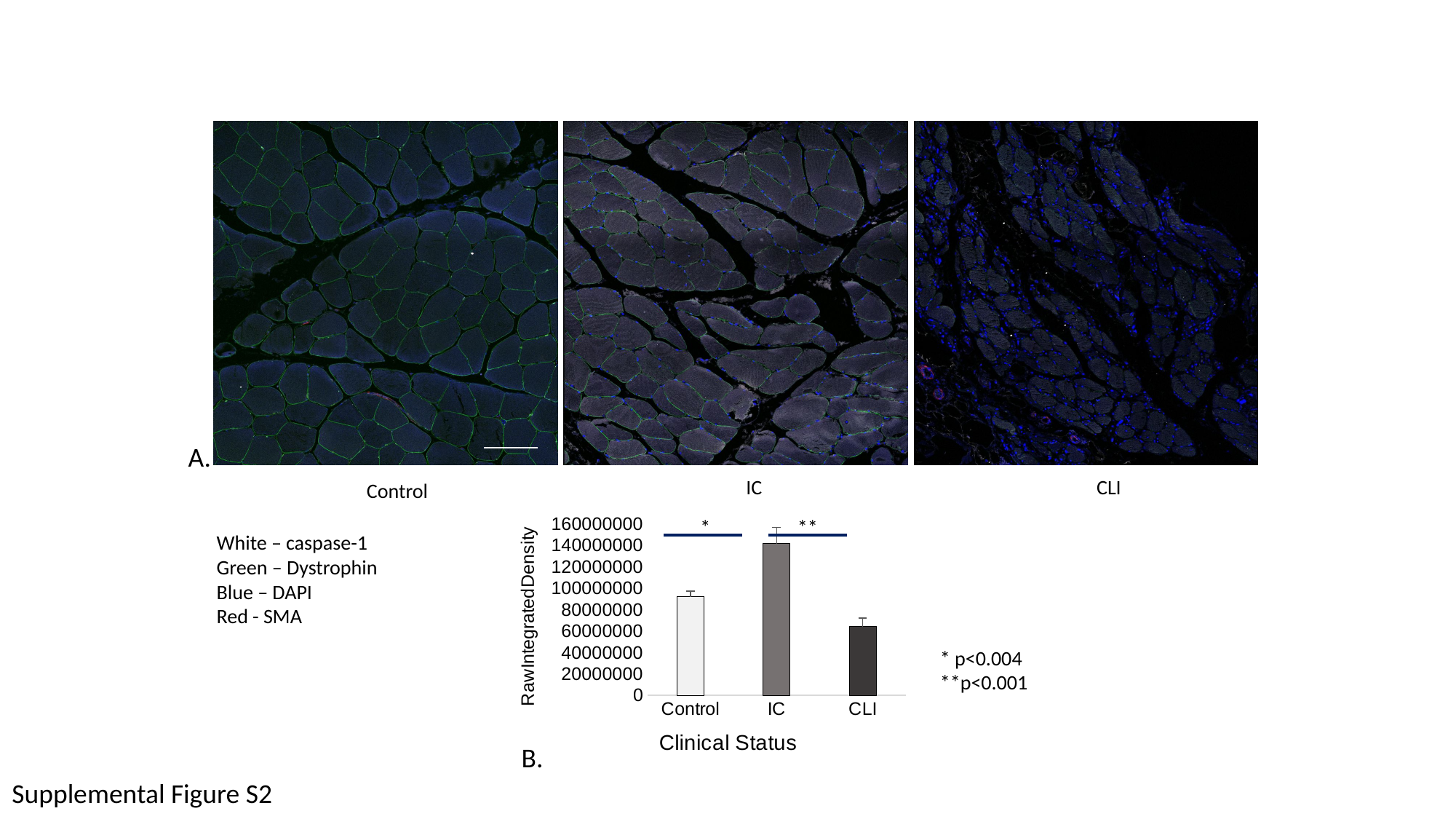

A.
IC
CLI
Control
### Chart
| Category | |
|---|---|
| Control | 91915662.462 |
| IC | 141564210.929 |
| CLI | 64229697.588 |*
**
White – caspase-1
Green – Dystrophin
Blue – DAPI
Red - SMA
* p<0.004
**p<0.001
B.
Supplemental Figure S2
