# Supplementary material for: Inflammatory Caspase Activity Mediates HMGB1 Release and Differentiation in Myoblasts Affected by Peripheral Arterial Disease
Source: Cells. 2022 Mar 30;11(7):1163. doi: 10.3390/cells11071163 (PMC8997414; doi:10.3390/cells11071163)
Supplement: Supplementary file 1 [file cells-11-01163-s001.zip › SUPPLEMENTAL MATERIALS.pdf]

SUPPLEMENTAL MATERIALS

Supplemental Figure S1 – Graphic demonstrating source of muscle tissue from human samples, and processing techniques to isolate myoblasts

Supplemental Figure S2 – Caspase-1 expression is elevated in patients with intermittent claudication. Patients undergoing surgery for either non-ischemic vascular disease (N= 13), intermittent claudication (N=14), or critical limb ischemia (N=17) were the sources of muscle tissue which was harvested using core-needle biopsy. Sections were stained for caspase-1, dystrophin to visualize the muscle outline, DAPI for nuclei and smooth muscle actin to identify arterioles. Expression of caspase-1 measured using mean fluorescence intensity was significantly higher in muscle harvested from patients with intermittent claudication compared to the other two groups (**$p<0.01$).
